# Association of Adoption of Transcarotid Artery Revascularization With Center-Level Perioperative Outcomes

**DOI:** 10.1001/jamanetworkopen.2020.37885

**Published:** 2021-02-22

**Authors:** Jesse A. Columbo, Pablo Martinez-Camblor, A. James O’Malley, David H. Stone, Vikram S. Kashyap, Richard J. Powell, Marc L. Schermerhorn, Mahmoud Malas, Brian W. Nolan, Philip P. Goodney

**Affiliations:** 1 Section of Vascular Surgery, Dartmouth-Hitchcock Medical Center, Geisel School of Medicine at Dartmouth, Lebanon, New Hampshire; 2 Dartmouth Institute for Health Policy and Clinical Practice, Hanover, New Hampshire; 3 Department of Biomedical Data Science, Geisel School of Medicine at Dartmouth, Hanover, New Hampshire; 4 Vascular Center, Harrington Heart & Vascular Institute, University Hospitals Cleveland Medical Center, Case Western Reserve University, Cleveland, Ohio; 5 Division of Vascular and Endovascular Surgery, Beth Israel Deaconess Medical Center, Harvard Medical School, Boston, Massachusetts; 6 Division of Vascular and Endovascular Surgery, University of California, San Diego Health, San Diego; 7 Section of Vascular Surgery, Maine Medical Center, Portland

## Abstract

**Importance:**

Transcarotid artery revascularization (TCAR) may serve as a safer alternative to carotid endarterectomy (CEA) for certain patients with carotid artery stenosis.

**Objective:**

To determine the center-level association of TCAR adoption with overall perioperative outcomes for TCAR and CEA combined at centers performing both procedures.

**Design, Setting, and Participants:**

This comparative-effectiveness research was conducted with a difference-in-difference analysis using retrospective data from 2015 to 2019 from the Vascular Quality Initiative registry, a consortium of more than 400 centers in North America. Included patients underwent TCAR or CEA for carotid artery stenosis. Patients who underwent transfemoral carotid stenting were excluded. Data were analyzed from December 2019 through August 2020.

**Exposures:**

Center-level adoption of TCAR vs not.

**Main Outcomes and Measures:**

The rate of major adverse cardiovascular events (MACE), a composite of in-hospital stroke, myocardial infarction, or death at 30 days, was measured.

**Results:**

Among 86 027 patients who underwent revascularization for carotid artery stenosis, 7664 patients (8.9%) underwent TCAR (mean [SD] age, 73.1 [9.6] years; 2788 [36.4%] women; 6938 White patients [90.6%]; and 3741 patients with symptoms [48.8%]) and 78 363 patients (91.1%) underwent CEA (mean [SD] age, 70.6 [9.2] years; 30 928 [39.5%] women; 70 663 White patients [90.2%]; and 37 883 patients with symptoms [48.3%]). The number of centers performing both TCAR and CEA increased from 15 centers in 2015 to 247 centers in 2019, a more than 16-fold increase. The proportion of all carotid procedures that were TCARs increased from 90 of 12 276 (0.7%) in 2015 to 2718 of 15 956 (17.0%) in 2019, a 24-fold increase. Overall, the crude rate of MACE was similar for TCAR and CEA (178 patients [2.3%] after TCAR vs 1842 patients [2.4%] after CEA; *P* = .91). However, the rate of MACE over time decreased for CEA (406 of 16 404 patients [2.5%] in 2015 vs 189 of 10 097 patients [1.9%] in 2019; *P* for trend < .001). The rate of MACE over time decreased for TCAR as well, but the change was not statistically significant (4 of 128 patients [3.1%] in 2016 vs 59 of 2718 patients [2.2%] in 2019; *P* for trend = .07). Difference-in-difference analysis demonstrated that centers that adopted TCAR had a 10% decrease in the likelihood of MACE at 12 months after TCAR adoption vs if those centers had continued to perform CEA alone (odds ratio, 0.90; 95% CI, 0.81-0.99; *P* = .04).

**Conclusions and Relevance:**

This comparative-effectiveness study of a cohort of patients who underwent TCAR or CEA found that availability of TCAR at a hospital was associated with a decrease in the likelihood of perioperative MACE after carotid revascularization.

## Key Points

**Question:**

Is the adoption of transcarotid artery revascularization (TCAR) associated with a center’s overall rate of perioperative cardiovascular events after carotid revascularization?

**Findings:**

In this comparative-effectiveness study of 7664 patients who underwent TCAR and 78 363 patients who underwent carotid endarterectomy at 469 centers in North America, centers that adopted TCAR had a lower likelihood of perioperative cardiovascular events than if the centers had not adopted the new procedure.

**Meaning:**

These findings suggest that the adoption of TCAR as an option for selected patients is associated with an improvement in perioperative outcomes for carotid revascularization overall at centers performing the procedure.

## Teaser

This comparative effectiveness research compares the rates of adverse events for patients who underwent revascularization for carotid artery stenosis in centers that adopted transcarotid artery revascularization vs those that did not.

## Introduction

In 2015, the Food and Drug Administration (FDA) approved a new procedure to treat patients with carotid artery stenosis: transcarotid artery revascularization with stenting (TCAR).^1^ This procedure is performed via a low neck incision with a direct carotid arterial puncture and uses flow-reversal embolic protection before manipulation of the carotid atherosclerotic lesion.^2,3^ These features of TCAR remove the need to traverse the aortic arch and carotid lesion prior to embolic protection that is necessary with transfemoral carotid stenting, and early results with TCAR have been favorable.^4,5,6^

This procedure may be particularly valuable in patients with challenging anatomy, such as a high carotid artery bifurcation or a carotid lesion close to the skull base, that would make traditional carotid endarterectomy (CEA) technically difficult.^2,3^ For these patients, TCAR provides a therapeutic alternative that may be associated with a lower risk profile than CEA. Therefore, we hypothesized that adoption of TCAR as a procedural option for patients may be associated with lower rates of perioperative adverse events for carotid revascularization overall (ie, TCAR and CEA combined).

To test this hypothesis, we employed a difference-in-difference analysis to determine the change in perioperative outcomes over time at centers adopting TCAR vs if those centers had continued to perform CEA alone. The difference-in-difference analysis captures the combined associations of the ability of a proceduralist to select the best procedure for each patient and of the potential outcomes for each patient under each procedure.^7^

## Methods

The institutional review board at Dartmouth-Hitchcock Medical Center approved this comparative-effectiveness study and determined that the need for specific informed consent could be waived because all data were deidentified prior to analysis. The study was conducted in accordance with the Strengthening the Reporting of Observational Studies in Epidemiology (STROBE) reporting guideline.

### Data Sources

We studied patients treated with TCAR or CEA who were entered into the Vascular Quality Initiative from 2015 to 2019. The Vascular Quality Initiative is a national quality improvement registry maintained by the Society for Vascular Surgery, a collection of modules capturing granular clinical information on 12 types of vascular procedures performed in more than 400 centers in North America.^8^ The TCAR Surveillance Project was initiated to capture and monitor outcomes after TCAR treatment using the carotid stenting registry operated by the Vascular Quality Initiative, which contains information on patients undergoing TCAR and transfemoral carotid artery stenting. Audits of device sales records indicate that the Surveillance Project captures more than 95% of TCAR procedures.^1^ The Vascular Quality Initiative also captures CEA procedures widely via a separate registry. Approximately 10% of all patients undergoing CEA in the United States undergo their procedure in a Vascular Quality Initiative center.^9,10^

### Patients

We studied patients captured by the Vascular Quality Initiative who underwent TCAR or CEA from January 2015, which was the year of TCAR FDA approval, through August 2019, which was the date of most recent data availability. For the estimation of procedure use rates, we included all patients. For outcome analysis, we excluded patients who underwent transfemoral carotid stenting, as this technique has been found to have approximately 2-fold the rate of perioperative stroke compared with CEA for patients presenting with focal neurologic symptoms.^11^ Furthermore, early results comparing transfemoral carotid stenting to TCAR have found superior results associated with TCAR.^12^ Nonetheless, we conducted a sensitivity analysis of perioperative events, including transfemoral carotid stenting, but excluded these from the primary analyses; results are available in the eAppendix 2 in the Supplement.

We excluded patients who underwent TCAR or CEA for indications other than atherosclerotic or neointimal hyperplastic disease (eg, for traumatic injury or dissection). We also excluded patients who underwent carotid revascularization with a concomitant procedure (eg, TCAR as part of an intracranial procedure or CEA at the time of coronary artery bypass grafting) and patients for whom the indication for the procedure was unknown (eg, performed for focal neurologic symptoms vs asymptomatic stenosis).

### Primary Exposure

Our primary exposure of interest was the state of TCAR adoption, measured at the center level. Centers were classified as having adopted TCAR once they started performing the new procedure.

### Analyses of TCAR Use Rates

In addition to categorizing centers as those that had adopted TCAR vs those that had not, we measured the rate of TCAR adoption over time. This was done by calculating the proportion of TCAR procedures out of the total carotid revascularizations performed at each center (ie, No. of TCAR procedures/[No. of TCAR + CEA + transfemoral stenting procedures]) by year. Participation in the Vascular Quality Initiative is voluntary, and centers may choose to opt in or out at any time. Centers may also participate in the registry for TCAR and transfemoral carotid stenting (ie, the carotid stenting registry) without participating in the registry for CEA. We calculated the proportion of procedures at 3-month intervals, including all centers participating in both registries during that 3-month interval. This allowed us to capture centers entering and exiting the Vascular Quality Initiative over the 5-year study period.

### Primary Outcome

Our primary outcome of interest was major adverse cardiovascular events (MACE), defined as a composite measure of in-hospital stroke, including ocular and cortical events; myocardial infarction, defined as clinical symptoms, electrocardiographic changes, or troponin elevation; or death within 30 days. Secondary outcomes included the individual outcomes in the composite measure, cranial nerve injury, transient ischemic attack, reperfusion syndrome, dysrhythmia, acute heart failure, operative time, reoperation or additional procedures to control bleeding, hospital length of stay of more than 1 day, and technical failure of the intended procedure. All outcomes were measured in hospital, with the exception of 30-day mortality, which was calculated using the Social Security Death Index.

### Statistical Analysis

We sought to estimate the association of TCAR adoption with the rate of perioperative MACE after carotid revascularization overall (ie, for TCAR and CEA combined). To do this, we conducted a difference-in-difference analysis, which assessed the center-level likelihood of perioperative MACE after TCAR and CEA combined and estimated the association of TCAR adoption with perioperative events vs what would have been expected if that center had not adopted TCAR^7^ (eAppendix 1 in the Supplement). We hypothesized that TCAR would be associated with improved outcomes among certain patients. Therefore, we created a difference-in-difference model that allowed for the selection of different procedures for specific patients. This analysis estimated the center-level association of TCAR adoption with likelihood of perioperative MACE vs the counterfactual (ie, if that center had not adopted TCAR) while allowing for proceduralists to select which intervention was performed on each patient; a better center would be associated with an increased likelihood that patients underwent the procedure that was best for them (ie, a better selection outcome).

We began by calculating the monthly rate of MACE for each center after CEA. Next, we created an indicator variable for when TCAR was first performed (ie, indicating TCAR adoption), if ever performed, for each center. Thereafter, the rate of MACE at TCAR-adopting centers was the rate of MACE for TCAR and CEA combined. The rate of MACE at centers not performing TCAR, including procedures completed at centers before TCAR adoption, was the rate for CEA alone. Centers performing TCAR but not participating in the CEA registry were excluded from this analysis, as their combined rate of MACE was unknown. We adjusted for all covariates listed in Table 1 and for the associations of center in the final regression model of MACE. The resultant model compared the relative change in the overall rate of MACE at centers performing TCAR and CEA with that of centers performing CEA alone; the latter centers had less ability to align patients with the procedure that was optimal for them, because these centers were unable to assign any patients to TCAR.

**Table 1.** Patient Characteristics

Characteristic	No. (%)^a^		
	TCAR (n = 7664)	CEA (n = 78 363)	*P* value
Age, mean (SD), y	73.1 (9.6)	70.6 (9.2)	<.001
Women	2788 (36.4)	30 928 (39.5)	<.001
Obesity (BMI > 30)	2587 (33.9)	26 884 (34.4)	.47
Missing	39 (<0.1)	89 (0.1)	NA
Race			
White	6938 (90.6)	70 663 (90.2)	.30
Black	338 (4.4)	3770 (4.8)	.12
Asian	61 (0.8)	856 (1.1)	.18
Pacific Islander	5 (0.1)	63 (0.1)	.81
American Indian or Alaskan Native	42 (0.6)	177 (0.2)	<.001
Other	273 (3.6)	2782 (3.6)	.44
Missing	7 (<0.1)	52 (0.1)	NA
Hispanic or Latino	290 (3.8)	2789 (3.6)	.32
Symptoms	3741 (48.8)	37 883 (48.3)	.44
CAD	3876 (50.6)	20 757 (26.5)	<.001
Missing	3 (<0.1)	51 (0.1)	NA
CHF	1356 (17.7)	8669 (11.1)	<.001
Missing	4 (<0.1)	28 (<0.1)	NA
Coronary revascularization	3066 (40.0)	26 976 (34.4)	<.001
Hypertension	6951 (90.8)	69 911 (89.2)	<.001
Missing	5 (<0.1)	7 (<0.1)	NA
COPD	2076 (27.1)	18 104 (23.1)	<.001
Home oxygen	275 (3.6)	1714 (2.2)	<.001
Missing	5 (<0.1)	38 (<0.1)	NA
Diabetes	2925 (38.2)	28 430 (36.3)	.001
Missing	1 (<0.1)	16 (<0.1)	NA
CKD (creatinine >1.7 mg/dL)	471 (6.2)	4386 (5.6)	.05
Smoking status			
Active	1705 (22.3)	20 051 (25.6)	<.001
Prior	3970 (51.9)	38 208 (48.8)	<.001
Missing	9 (<0.1)	32 (<0.1)	NA
Prior carotid procedure			
Ipsilateral	1222 (15.9)	1581 (2.0)	<.001
Contralateral	1167 (15.2)	10 801 (13.8)	.001
Preoperative medications			
Aspirin	6840 (89.3)	65 536 (83.7)	<.001
Missing	1 (<0.1)	21 (<0.1)	NA
P2y12 inhibitor	6627 (86.5)	27 084 (34.6)	<.001
Missing	3 (<0.1)	25 (<0.1)	NA
Dual antiplatelet	6042 (78.9)	22 610 (28.9)	<.001
Missing	3 (<0.1)	25 (<0.1)	NA
Statin	6822 (89.0)	65 455 (83.6)	<.001
Missing	1 (<0.1)	18 (<0.1)	NA
β blocker	4406 (57.5)	42 707 (54.5)	<.001
Missing	3 (<0.1)	41 (0.1)	NA
Anticoagulation	1102 (14.4)	5549 (7.1)	<.001
Missing	4 (<0.1)	151 (0.2)	NA
ACE inhibitor	4084 (53.3)	41 559 (53.1)	.66
Missing	5 (<0.1)	31 (<0.1)	NA
Functional status			
Ambulatory	7242 (96.2)	77 152 (98.6)	<.001
Wheelchair	271 (3.6)	1018 (1.3)	<.001
Severe mobility impairment^b^	12 (0.2)	52 (0.1)	.009
Missing	139 (0.2)	141 (0.2)	NA
Insurance			
Medicare	5024 (65.7)	44 112 (56.3)	<.001
Medicaid	216 (2.8)	2974 (3.8)	<.001
Private	2339 (30.6)	29 828 (38.1)	<.001
Non-US insurance or none	65 (0.9)	1400 (1.8)	<.001
Missing	20 (<0.1)	49 (0.1)	NA

Abbreviations: ACE, angiotensin-converting enzyme; BMI, body mass index (calculated as weight in kilograms divided by height in meters squared); CAD, coronary artery disease; CEA, carotid endarterectomy; CHF, congestive heart failure; CKD, chronic kidney disease; COPD, chronic obstructive pulmonary disease; NA, not applicable; TCAR, transcarotid artery revascularization.

SI conversion: To convert creatinine to μmol/L, multiply by 88.4.

^a^ Percentages are calculated out of the total known values for each variable. There was less than 5% missing data for each variable.

^b^ Defined as being unable to get out of bed unassisted.

Patient characteristics and outcomes were calculated out of the known (ie, nonmissing) values for each variable. There was less than 5% missing information for each variable. We summarized continuous measures with means and SDs or medians with interquartile ranges (IQRs) as appropriate. We reported proportions as percentages. We first calculated the rate of MACE for all patients who underwent TCAR or CEA and met our inclusion criteria. We then created crude and adjusted logistic regression models reporting odds ratios (ORs), with 95% CIs (95% CI), of MACE for TCAR vs CEA.

We included all characteristics in Table 1 in the adjusted models. Additionally, we adjusted for the associations of the center performing the procedure and for calendar time. Patients with missing data were excluded from the final regression. The number of missing observations and the patients included in each analysis are described in the eFigure in the Supplement. Race and ethnicity were reported according to the classifications designated by the registry.

Statistical analyses were conducted using R statistical software version 3.3.1 (R Project for Statistical Computing) and Stata statistical software version 15 (StataCorp) from December 2019 through August 2020. *P* values were 2-sided, a *P* < .05 was considered statistically significant.

## Results

### Patient and Procedural Characteristics

Among 86 027 patients who underwent treatment for carotid artery stenosis, 7664 patients (8.9%) underwent TCAR and 78 363 patients (91.1%) underwent CEA (Table 1). Patients undergoing TCAR, compared with those undergoing CEA, were older (mean [SD] age, 73.1 [9.6] years vs 70.6 [9.2] years; *P* < .001) and less likely to be women (2788 [36.4%] women vs 30 928 [39.5%] women; *P* < .001); similar proportions were White (6938 patients [90.6%] vs 70 663 patients [90.2%]; *P* = .30). Revascularization was performed for neurologic symptoms in 3741 patients who underwent TCAR (48.8%) and 37 883 patients who underwent CEA (48.3%) (*P* = .44). Comorbid conditions, preoperative medications, and other clinical characteristics were typical for this patient population.

Carotid revascularization was performed for severe carotid stenosis (ie, ≥70% carotid artery stenosis) in 6495 TCAR procedures (86.0%) and 61 592 (82.0%) CEA procedures (Table 2). Patients who underwent TCAR were deemed to be at high risk for surgical treatment owing to anatomic indications (3744 patients [49.1%]) or medical indications (4220 patients [55.4%]). Most procedures were performed on an elective basis and under general anesthesia.

**Table 2.** Periprocedural Characteristics and Outcomes

Variable	No. (%)^a^		
	TCAR (n = 7664)	CEA (n = 78 363)	*P* value
Periprocedural characteristic			
High risk			
Anatomic	3744 (49.1)	3429 (5.0)	<.001
Missing	44 (0.1)	146 (0.2)	NA
Medical	4220 (55.4)	NA	NA
Missing	44 (0.1)	NA	NA
Refused for surgery	1690 (22.4)	NA	NA
Missing	119 (0.2)	NA	NA
Degree of stenosis, %			
<50	240 (3.2)	2752 (3.7)	.03
50-69	811 (10.8)	10 553 (14.1)	<.001
70-79	2363 (31.3)	26 354 (35.2)	<.001
80-99	3965 (52.5)	34 158 (45.6)	<.001
Occluded	167 (2.2)	1080 (1.4)	<.001
Missing	118 (0.2)	3466 (4.4)	NA
Urgency			
Elective	6902 (90.1)	68 609 (87.6)	<.001
Urgent	747 (9.8)	9285 (11.9)	<.001
Emergent	15 (0.2)	448 (0.6)	<.001
Missing	0	21 (<0.1)	NA
ASA class			
1	61 (0.8)	492 (0.6)	.09
2	336 (4.4)	3319 (4.2)	.52
3	5058 (66.3)	58 173 (74.4)	<.001
4	2167 (28.4)	16 234 (20.8)	<.001
5	3 (<0.01)	24 (0.03)	.95
Missing	39 (<0.1)	121 (0.2)	NA
Anesthetic type			
General	6097 (79.6)	72 425 (92.4)	<.001
Local or regional	1561 (20.4)	5926 (7.6)	<.001
Missing	6 (<0.1)	12 (<0.1)	NA
Procedural anticoagulation	7548 (98.8)	77 615 (99.1)	.02
Missing	24 (<0.1)	20 (<0.1)	NA
Protamine	5943 (79.6)	55 565 (71.0)	<.001
Missing	193 (0.2)	45 (0.1)	NA
Balloon angioplasty after stenting	4217 (55.7)	NA	NA
Missing	93 (0.1)	NA	NA
Outcomes			
Stroke, MI, or 30-d death	178 (2.3)	1842 (2.4)	.91
Stroke or 30-d death	149 (1.9)	1368 (1.7)	.22
Stroke	106 (1.4)	987 (1.3)	.39
Ipsilateral stroke	92 (1.2)	749 (1.0)	.04
TIA	55 (0.7)	408 (0.5)	.03
In-hospital death	34 (0.4)	221 (0.3)	.02
30-d death	61 (0.8)	493 (0.6)	.10
MI	37 (0.5)	554 (0.7)	.03
Reperfusion syndrome	16 (0.2)	124 (0.2)	.37
Dysrhythmia	123 (1.6)	1062 (1.4)	.08
Acute heart failure	35 (0.5)	302 (0.4)	.39
Cranial nerve injury	19 (0.2)	2041 (2.6)	<.001
Operative time, median (IQR), min	67 (53-86)	108 (85-137)	<.001
Reoperation for bleeding	100 (1.3)	1238 (1.6)	.07
Length of stay >1 d	2214 (28.9)	24 834 (31.7)	<.001
Technical failure	39 (0.5)	NA	NA

Abbreviations: ASA, American Society of Anesthesiologists; CEA, carotid endarterectomy; IQR, interquartile range; MI, myocardial infarction; NA, not applicable; TCAR, transcarotid artery revascularization; TIA, transient ischemic attack.

^a^ Percentages are calculated out of the total known values for each variable. There was less than 5% missing data for each variable.

### TCAR Use Rates

**Figure 1.**
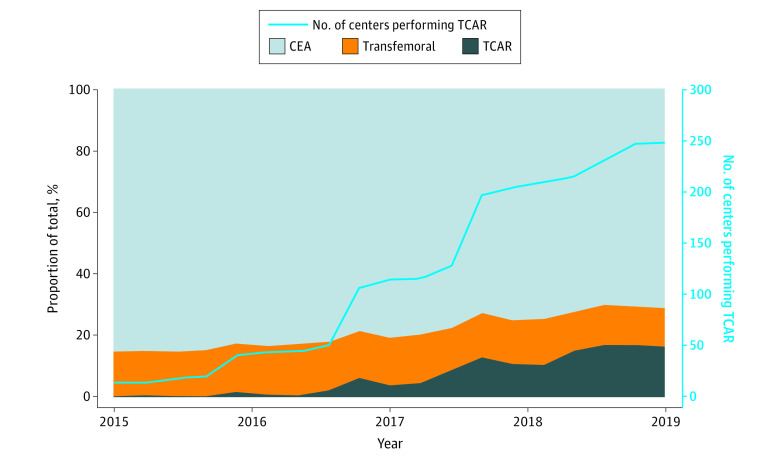
Proportion of Carotid Revascularization Procedures Performed Proportions of procedures were calculated for centers participating in both the carotid endarterectomy (CEA) and carotid artery stenting registries at 3-month intervals. TCAR indicates transcarotid artery revascularization with stenting.

In the Vascular Quality Initiative registry, we found 15 centers that performed both TCAR and CEA in 2015. This increased to 247 centers in 2019, a more than 16-fold increase (Figure 1; eTable in the Supplement). The use of TCAR out of the total carotid revascularization procedures increased from 90 of 12 276 procedures (0.7%) in 2015 to 2718 of 15 956 procedures (17.0%) in 2019, a 24-fold increase over 5 years.

### Center-Level MACE by TCAR Adoption Status

Overall, MACE occurred in 178 patients receiving TCAR (2.3%) and 1842 patients receiving CEA (2.4%; *P* = .91) (Table 2). The rate of MACE decreased over time for CEA (406 of 16 404 patients [2.6%] in 2015 vs 189 of 10 097 patients [1.9%] in 2019; *P* for trend = <.001). The rate of MACE over time decreased for TCAR as well, but the change was not statisically significnat (4 of 128 patients [3.1%] in 2016 vs 59 of 2718 patients [2.2%] in 2019; *P* for trend = .07). The rate of perioperative stroke was similar in the 2 groups, with 104 patients who received TCAR (1.4%) and 987 patients who received CEA (1.3%) experiencing stroke (*P* = .39). Cranial nerve injury was less common among patients receiving TCAR compared with those receiving CEA (19 patients [0.2%] vs 2041 patients [2.6%]; *P* < .001). Median (IQR) operative time was shorter for TCAR (67 [53-86] minutes vs 108 [85-137] minutes; *P* < .001). The rate of technical failure was low in both groups (TCAR: 39 failures [0.5%]; CEA: 0 failures ).

**Figure 2.**
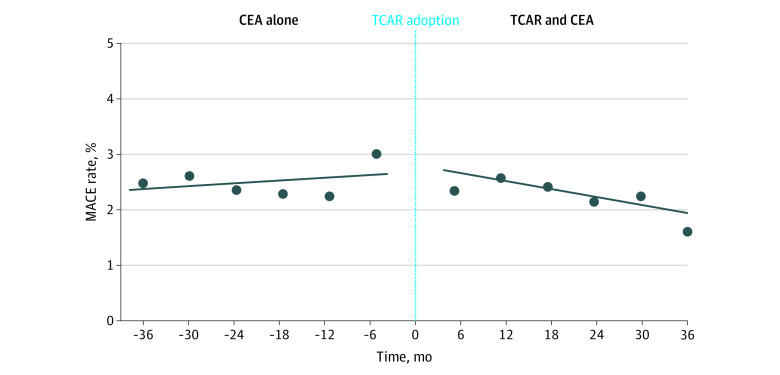
Rate of Perioperative Major Adverse Cardiovascular Events (MACE) Rate of MACE prior to the adoption of transcarotid artery revascularization (TCAR) represents the rate for carotid endarterectomy (CEA) alone at centers that never adopted TCAR or had not yet adopted TCAR. For centers adopting TCAR, the rate after TCAR adoption represents the rate of MACE for CEA and TCAR combined. Centers performing TCAR alone are not represented.

The difference-in-difference analysis estimated the center-level likelihood of MACE after TCAR and CEA combined vs the likelihood of MACE that would have been expected if that center had not adopted TCAR (Figure 2). This analysis found that TCAR adoption was associated with a 10% reduction in the risk of MACE at 12 months compared with what would have been expected had the center not adopted TCAR (adjusted OR, 0.90; 95% CI, 0.81-0.99; *P* = .04; per-month OR, 0.991; 95% CI, 0.983-0.999; *P* = .04).

### Patient-Level MACE by Procedure Type and Symptom Status

**Figure 3.**
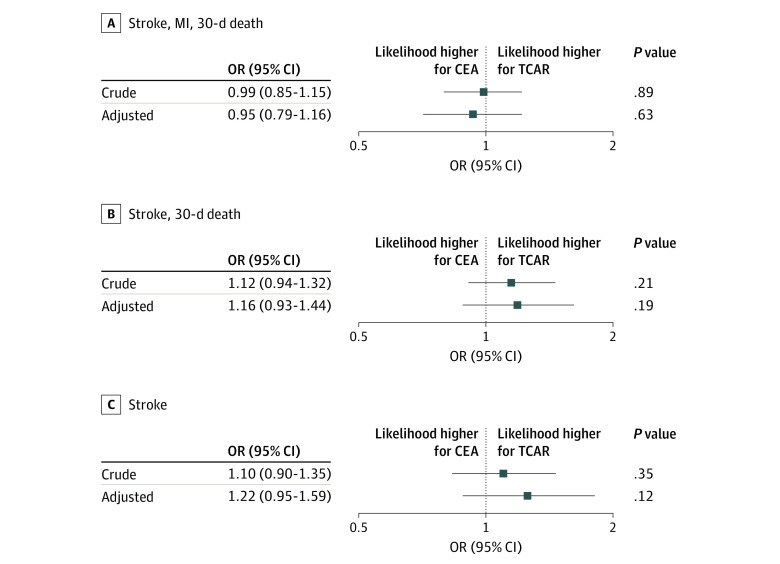
Outcomes After Procedures All characteristics in Table 1 were included in the adjusted regression model. CEA indicates carotid endarterectomy; MI, myocardial infarction; OR, odds ratio; TCAR, transcarotid artery revascularization with stenting.

The crude OR of MACE for TCAR vs CEA was 0.99 (95% CI, 0.85-1.15; *P* = .89) (Figure 3). The adjusted OR was similar, at 0.95 (95% CI, 0.79-1.16; *P* = .63). Similar results were found for the ORs for stroke or 30-day death and for stroke alone.

We performed a subanalysis for the OR of MACE stratified by presenting neurologic symptoms (ie, symptomatic vs asymptomatic carotid stenosis). We found that perioperative outcomes were similar between TCAR and CEA among patients with symptoms and patients without symptoms. Among patients with symptoms, the crude OR was 1.02 (95% CI, 0.84-1.24; *P* = .84) and the adjusted OR was 0.93 (95% CI, 0.73-1.19; *P* = .56). Among patients without symptoms, the crude OR was 0.91 (95% CI, 0.70-1.29; *P* = .60) and the adjusted OR was 0.96 (95% CI, 0.69-1.34; *P* = .81).

## Discussion

This comparative-effectiveness study found that among more than 85 000 patients treated across 469 centers, TCAR adoption was associated with a 10% lower likelihood of perioperative MACE after TCAR and CEA combined vs if the center had continued to perform CEA alone. Furthermore, adjusted logistic regression models comparing TCAR with CEA found that TCAR had a perioperative likelihood of MACE that was similar to the results obtained with CEA. These findings suggest that the adoption of TCAR as an option for carotid revascularization, making it available for select patients for whom it is the best treatment option, is associated with better overall performance.

It was our hypothesis that the introduction of a new procedure type would be associated with improved overall outcomes for a center, with increased flexibility to align patients with the procedure best suited to them. This notion has been put forth previously in the adoption of treatments for aortic valve disease. As a minimally invasive alternative to traditional surgical aortic valve replacement, transcatheter aortic valve replacement has demonstrated favorable results, with shorter hospital lengths of stay, decreased rates of stroke, and decreased rates of mortality compared with surgical aortic valve replacement.^13^ Much like the data we found for TCAR and CEA, other investigators have found that a center’s adoption of transcatheter aortic valve replacement was also associated with improvements in outcomes for patients undergoing surgical aortic valve replacement at the same institution.^14^

Will outcomes always improve when a new procedure is introduced? We believe 2 important factors may be required for a new procedure to be associated with improvements in overall perioperative outcomes for a disease process at an institution. First, the procedure must be associated with a low perioperative event rate for at least some of the patients who are treated. In 2 studies from 2020^3,15^ investigators found that TCAR was associated with low perioperative adverse event rates and that these rates are similar among centers that recently adopted TCAR compared with centers with more experience. Second, the procedure must be correctly aligned with the patient group expected to benefit from the new technology. The FDA approved TCAR for use in patients deemed to be at high risk for surgery for anatomic reasons (eg, reoperative field) or medical reasons (eg, severe heart failure).^1,15^ In these patients, TCAR may provide an alternative to CEA that is lower risk. We found that TCAR was adopted widely during the study interval, representing 17.0% of procedures in 2019, and rates of perioperative MACE for the 2 procedures decreased over time. However, centers adopting TCAR had a 10% lower likelihood of perioperative MACE after carotid revascularization overall (ie, TCAR and CEA combined). This finding suggests that this new technology may have allowed proceduralists to select patients for whom TCAR may be superior to CEA, while still performing CEA on patients for whom that procedure was appropriate. Comparing TCAR directly with CEA using an adjusted regression model, we found that TCAR and CEA had a similar likelihood of perioperative MACE. Overall, while observational in nature, these data imply that both TCAR and CEA may be reasonable treatment choices for patients undergoing carotid revascularization and providers may be able to choose the modality they feel best aligns with the patient’s clinical presentation and anatomy.

Our study comparing TCAR and CEA builds on published work of other authors. Single-arm clinical trials from 2015^6^ and 2020^15^ found low rates of perioperative MACE for TCAR. Studies comparing TCAR with CEA using logistic regression have found similar rates of perioperative MACE associated with the 2 procedures.^5^ Our addition of a center-level investigation incorporating a difference-in-difference analysis provides further insight on the associations of the adoption of TCAR with outcomes among patients who undergo carotid revascularization. We designed the difference-in-difference analysis so it would not be restricted to an identical group of patients. This allowed for an examination of the association of TCAR with patient selection and of the association of this selection with carotid revascularization outcomes, suggesting that different patients may benefit from different procedures, as we hypothesized. These findings add to the growing body of research that supports adoption of TCAR as an option for carotid revascularization.

### Limitations

This study has several limitations. It is possible that centers that adopted TCAR differ from centers that did not adopt this procedure (eg, TCAR adoption may be a surrogate for a higher-quality center). We accounted for this in the following ways. First, the results from centers eventually adopting TCAR were compared with those centers’ own results prior to TCAR adoption (ie, their results for CEA only). This allowed for a direct comparison within a center. Second, we included center as a covariate in the regression model, which adjusts for the differences in results across centers within the CEA alone and TCAR plus CEA groups. In addition, while the vast majority of TCAR procedures are captured by the Vascular Quality Initiative, the registry includes a much smaller proportion of CEA procedures performed nationally, which may limit generalizability to centers that do not participate in the Vascular Quality Initiative registry. Data from the Vascular Quality Initiative is self-reported by centers and, as such, may be associated with reporting bias. We chose a composite of stroke, myocardial infarction, and death as our primary outcome because of the perioperative focus for this study. We performed comparative analyses using stroke alone and stroke or death and found no significant difference between TCAR and CEA. The FDA approved TCAR in 2015, and as such, long-term results are not yet known.

## Conclusions

This comparative-effectiveness study found that centers adopting TCAR had a 10% lower likelihood of perioperative MACE after carotid revascularization vs if they had continued to perform CEA alone, although TCAR and CEA have similar perioperative results. Adoption of TCAR as an option for carotid revascularization may allow proceduralists to align patients with the procedure best suited to them and is associated with an overall improvement in perioperative outcomes across all patients receiving TCAR and CEA at a center.

## Notes

Supplement. **eFigure.** Flow Diagram of Patients Included in the Study **eAppendix 1.** Difference-in-Difference Analysis **eTable.** Characteristics of Transcarotid Artery Revascularization Patients Over Time **eAppendix 2.** Results of Patients Who Underwent Transfemoral Carotid Stenting **eReferences**
