# Supplementary material for: Association of Adoption of Transcarotid Artery Revascularization With Center-Level Perioperative Outcomes
Source: JAMA Netw Open. 2021 Feb 22;4(2):e2037885. doi: 10.1001/jamanetworkopen.2020.37885 (PMC7900862; doi:10.1001/jamanetworkopen.2020.37885)
Supplement: Supplement. — eFigure. Flow Diagram of Patients Included in the Study eAppendix 1. Difference-in-Difference Analysis eTable. Characteristics of Transcarotid Artery Revascularization Patients Over Time eAppendix 2. Results of Patients Who Underwent Transfemoral Carotid Stenting eReferences [file jamanetwopen-e2037885-s001.pdf]

Supplemental Online Content

Columbo JA, Martinez-Camblor P, O'Malley AJ, et al. Association of adoption of transcarotid artery revascularization with center-level perioperative outcomes. *JAMA Netw Open*. 2021;4(2):e2037885. doi:10.1001/jamanetworkopen.2020.37885

eFigure. Flow Diagram of Patients Included in the Study
eAppendix 1. Difference-in-Difference Analysis
eTable. Characteristics of Transcarotid Artery Revascularization Patients Over Time
eAppendix 2. Results of Patients Who Underwent Transfemoral Carotid Stenting
eReferences

© 2021 Columbo JA et al. *JAMA Network Open*.

eFigure. Flow Diagram of Patients Included in the Study

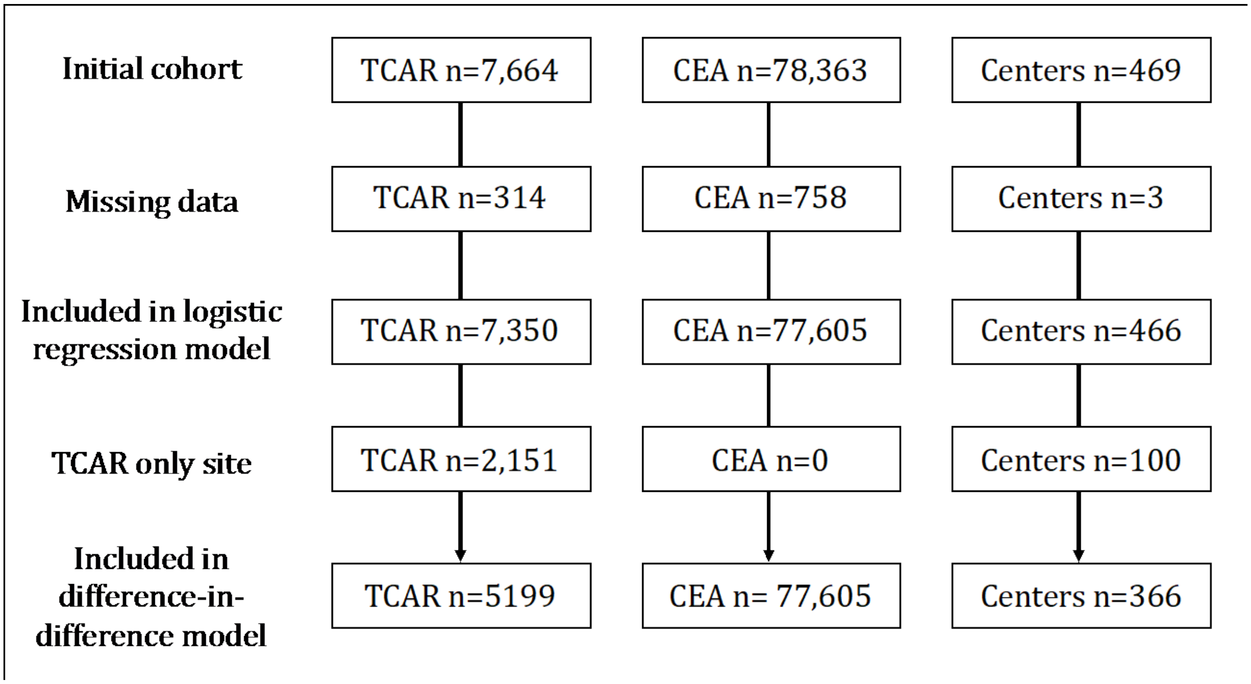

© 2021 Columbo JA et al. *JAMA Network Open*.

eAppendix 1. Difference-in-Difference Analysis

We conducted a difference-in-difference analysis to determine the effect of transcarotid artery revascularization (TCAR) on major adverse cardiovascular events (MACE) after carotid revascularization versus the counterfactual (e.g. if that center had not adopted TCAR).[1] Although both procedures treat carotid artery stenosis with the objective of reducing stroke, TCAR and carotid endarterectomy (CEA) are distinct procedures with differences in technical nuance for each. As such, some patients may be better suited for TCAR, and some for CEA. Therefore, we felt that it was important to allow for patient-procedure selection to occur as this is representative of real-world patient care.

We began by calculating the monthly rate of MACE for each center after CEA. Next, we created an indicator variable for when TCAR was first performed (e.g. indicating TCAR adoption), if ever performed, for each center. Thereafter, the rate of MACE at TCAR adopting centers was the rate of MACE for TCAR and CEA combined. The rate of MACE at centers not performing TCAR, including centers prior to TCAR adoption, was the rate for CEA alone. Centers performing TCAR but not participating in the CEA registry were excluded from this analysis as their combined rate of MACE was unknown. We adjusted for all covariates in Table 1, and for the effects of center in the final regression model of MACE. The resultant model compared the relative change in the overall rate of MACE at centers performing TCAR and CEA versus centers performing CEA alone.

© 2021 Columbo JA et al. *JAMA Network Open*.

**eTable.** Characteristics of Transcarotid Artery Revascularization Patients Over Time				
	2015-2016	2017	2018	2019
Variable	% (n=129)	% (n=1,374)	% (n=3,443)	% (n=2,718)
Age, years, mean (SD)	72.5 (9.6)	72.7 (9.7)	73.3 (9.1)	73.1 (9.0)
Female sex	31.2 (40)	36.0 (494)	36.8 (1,268)	36.3 (986)
Obesity (BMI >30)	37.9 (44)	33.3 (452)	33.9 (1,165)	34.1 (926)
Hispanic or Latino	5.6 (7)	4.2 (57)	3.7 (128)	3.6 (98)
Race				
American Indian or Alaskan Native	0	1.0 (14)	0.5 (17)	0.4 (11)
Asian	0.8 (1)	1.0 (11)	0.8 (27)	0.8 (22)
Black or African American	3.1 (4)	3.9 (54)	4.9 (170)	4.1 (110)
Pacific Islander	0	0	0.1 (3)	0.1 (2)
White	95.3 (121)	91.2 (1,259)	89.8 (3,091)	90.8 (2,466)
Other	0.8 (1)	2.6 (35)	3.9 (133)	3.8 (104)
Symptomatic	45.3 (58)	47.0 (646)	49.7 (1,710)	48.8 (1,327)
CAD	48.8 (62)	50.9 (699)	51.1 (1,757)	50.0 (1,358)
CHF	20.3 (26)	18.8 (258)	18.1 (623)	16.5 (449)
Coronary Revascularization	39.8 (51)	42.4 (582)	40.3 (1,389)	38.4 (1,044)
HTN	88.3 (113)	90.7 (1,246)	89.9 (3,095)	91.9 (2,496)
COPD	23.6 (30)	27.2 (374)	27.8 (955)	26.4 (717)
home oxygen	2.4 (3)	3.4 (47)	3.9 (133)	3.4 (92)
Diabetes	29.7 (38)	37.2 (511)	38.3 (1,319)	38.9 (1,056)
CKD (creatinine >1.7 mg/dL)	7.8 (10)	6.8 (94)	5.8 (198)	6.2 (169)
Smoking	25.2 (32)	23.3 (319)	26.8 (923)	26.0 (706)
Active	26.8 (34)	25.3 (347)	21.6 (744)	21.4 (580)
Prior	48.0 (61)	51.5 (706)	51.6 (1,774)	52.6 (1,428)
Prior Ipsilateral carotid procedure	20.3 (26)	18.9 (259)	15.8 (543)	14.5 (394)
Prior contralateral carotid procedure	10.9 (14	15.9 (218)	15.3 (528)	15.0 (407)
Preoperative medications				
Aspirin	85.9 (110)	90.0 (1,236)	89.1 (3,065)	89.3 (2,428)
P2y12 inhibitor	87.4 (111)	84.7 (1,164)	85.9 (2,956)	88.1 (2,395)
Dual antiplatelet	77.3 (99)	78.1 (1,073)	78.1 (2,686)	80.3 (2,183)
Statin	91.4 (117)	88.2 (1,212)	88.6 (3,051)	89.8 (2,441)
Beta blocker	55.9 (71)	56.1 (770)	57.3 (1,974)	58.5 (1,590)
Anticoagulation	9.4 (12)	14.3 (196)	14.5 (500)	14.5 (394)
ACE inhibitor	49.2 (63)	53.9 (740)	53.9 (1,856)	52.5 (1,425)
Functional status				
Ambulatory	93.6 (103	96.4 (1,294)	96.3 (3,252)	96.3 (2,592)
Wheelchair	5.5 (6)	3.6 (48)	3.6 (123)	3.5 (94)
Bedridden	1 (1)	0.1 (1)	0.1 (3)	0.3 (7)
Insurance				
Medicare	59.0 (72)	64.9 (890)	66.4 (2,279)	65.7 (1,782)

© 2021 Columbo JA et al. *JAMA Network Open*.

	Medicaid	0	3.5 (48)	2.5 (87)	3.0 (81)
	Private	39.3 (48)	31.0 (425)	30.3 (1,039)	30.5 (827)
	Non US, or none	1.6 (2)	1.0 (9)	0.9 (30)	0.9 (24)

Percentages are calculated out of the total known values for each variable. There was less than 5% missing data for each variable.
Legend: TCAR, transcarotid artery revascularization; CEA, carotid endarterectomy; BMI, body mass index; TIA, transient ischemic attack; CAD, coronary artery disease; CHF, congestive heart failure; HTN, hypertension; COPD, chronic obstructive pulmonary disease; CKD, chronic kidney disease.

© 2021 Columbo JA et al. *JAMA Network Open*.

eAppendix 2. Results of Patients Who Underwent Transfemoral Carotid Stenting

We performed a sensitivity analysis among patients who underwent transfemoral carotid artery stenting. The crude rate of MACE for patients undergoing transfemoral carotid stenting was 4.1%. The crude odds ratio (OR) of MACE for TCAR versus transfemoral carotid stenting was 0.56 (95% confidence interval (95CI): 0.46-0.67) and for CEA versus transfemoral carotid stenting was 0.56 (95CI: 0.50-0.63). The instrumental variable adjusted ORs were similar, and for TCAR versus transfemoral carotid stenting was 0.63 (95CI: 0.30-1.35), and for CEA versus transfemoral carotid artery stenting was 0.51 (95CI: 0.29-0.92).

© 2021 Columbo JA et al. *JAMA Network Open*.

eReferences

1. Dimick JB, Ryan AM. Methods for evaluating changes in health care policy: the difference-in-differences approach. *Jama.* 2014;312(22):2401-2402.

© 2021 Columbo JA et al. *JAMA Network Open*.
